# An Alginate-Based Hydrogel with a High Angiogenic Capacity and a High Osteogenic Potential

**DOI:** 10.1089/biores.2020.0010

**Published:** 2020-06-05

**Authors:** Anaïs Barre, Marie Naudot, Fanny Colin, Henri Sevestre, Louison Collet, Bernard Devauchelle, Stéphane Lack, Jean-Pierre Marolleau, Sophie Le Ricousse

**Affiliations:** ^1^ EA7516, CHIMERE, Jules Verne University of Picardie, Amiens, France.; ^2^ Les Laboratoires Brothier, Nanterre, France.; ^3^ Department of Pathology and Anatomy, Amiens University Medical Center, Amiens, France.; ^4^ EA4666 HEMATIM, Jules Verne University of Picardie, Amiens, France.; ^5^ Department of Maxillofacial Surgery, Amiens University Medical Center, Amiens, France.; ^6^ Facing Faces Institute, Amiens, France.; ^7^ Department of Hematology, Amiens University Medical Center, Amiens, France.

**Keywords:** alginate-based hydrogel, angiogenic and osteogenic capacities, ectopic animal model, endothelial cell, mesenchymal stem cell

## Abstract

In bone tissue engineering, autologous cells are combined with osteoconductive scaffolds and implanted into bone defects. The major challenge is the lack of post-implantation vascular growth into biomaterial. The objective of the present study was to develop a new alginate-based hydrogel that enhances the regeneration of bone defects after surgery. The viability of human bone marrow-derived mesenchymal stem cells (BM-MSCs) or human endothelial cells (ECs) cultured alone or together on the hydrogel was analyzed for 24 and 96 h. After seeding, the cells self-assembled and aggregated to form clusters. For functional validation, empty or cellularized hydrogel matrices were implanted ectopically at subcutaneous sites in *nude* mice. After 2 months, the matrices were explanted. Transplanted human cells were present, and we observed vessels expressing human von Willebrand factor (resulting from the incorporation of transplanted ECs into neovessels and/or the differentiation of BM-MSCs into ECs). The addition of BM-MSCs improved host vascularization and neovessel formation from human cells, relative to ECs alone. Although we did not observe bone formation, the transplanted BM-MSCs were able to differentiate into osteoblasts. This new biomaterial provided an appropriate three-dimensional environment for transplanted cells and has a high angiogenic capacity and an osteogenic potential.

## Introduction

Recent advances in tissue engineering owe their success largely to the development of novel biomaterial-based strategies that better mimic native tissue and organ structure.^1^ For bone reconstruction, the ideal scaffold must (1) promote cell survival, proliferation, and differentiation, (2) facilitate and enhance vascularization, (3) inhibit fibrous tissue formation, and (4) be able to integrate into the surrounding tissue. As a natural nonthrombogenic hydrogel with good biocompatibility and biodegradability, alginate has been used variously for cell delivery *in vitro* and *in vivo*,^2,3^ vascular regeneration,^4^ cardiac repair,^5^ and bone tissue engineering.^6^ The combination of potentially osteogenic cells with a bone-mimicking scaffold is a promising alternative approach to bone grafting. Although the treatment of bone defects using mesenchymal stem cells (MSCs) can effectively promote bone regeneration in human and animal models,^7^ the lack of blood vessels deprives the bone graft of nutritional support. Great effort has therefore gone into developing novel tissue engineering strategies that promote blood vessel formation and thus improve bone regeneration; in particular, neovascularization can be induced by cell-based techniques with the implantation of differentiating endothelial cells (ECs) or endothelial progenitor cells (EPCs).^8,10^

The three major barriers to successful cell-based therapy are the poor distribution, survival, and integration of donor cells,^11,12^ suggesting that more effective delivery strategies and/or biomaterials are needed.

In the present study, we developed a novel alginate-based hydrogel and analyzed its ability to promote neovascularization and bone formation after cell transplantation. We first analyzed the scaffold's effect on the viability of human MSCs isolated from bone marrow (BM-MSCs) and human ECs (derived from EPCs isolated from umbilical cord blood). Next, we tested the cellularized scaffold's osteogenic and angiogenic properties following subcutaneous implantation in mice. The effect of co-culturing and transplanting a mixture of BM-MSCs and ECs in the scaffold was compared with the scaffold+BM-MSCs alone, the scaffold+ECs alone, and the empty (i.e., cell-free) hydrogel. Bone formation was assessed histologically, and neovessel formation was evaluated using immunohistochemical techniques.

## Materials and Methods

### Scaffold production

Alginate-based hydrogel scaffolds were synthesized as described in the patent.^13^ Briefly, the hydrogel was prepared in two steps, under sterile conditions. First, nonwoven calcium alginate fiber (Algosteril^®^; Laboratoires Brothier, Nanterre, France) was mixed with a trisodium citrate solution (10 g/L; VWR chemicals) to initiate cross-linking and gel formation. Second, the gel was incubated with sodium alginate (1.5%; Kimica, Tokyo, Japan) to produce a macroporous scaffold.

### Isolation and expansion of human BM-MSCs and ECs

Human bone marrow was obtained from the iliac crest of seven healthy donors. The donors had provided their written informed consent to use the sample for research purposes. After centrifugation, the buffy coat layer was isolated and seeded in T175 culture flasks containing Minimum Essential Medium Eagle-alpha modification (Sigma–Aldrich, France) supplemented with 10% fetal bovine serum (FBS; Eurobio, France), 2 mM L-glutamine, 100 IU/mL penicillin, 100 mg/mL streptomycin (Eurobio), and 0.5 ng/mL basic fibroblast growth factor (PeproTech, France). The cells were cultured in a humidified 5% CO_2_ atmosphere at 37°C. Nonadherent cells were removed after 4 days, and fresh medium was added.

Umbilical cord blood was obtained from full-term neonates (from problem-free pregnancies and deliveries) after the provision of written informed consent by the mother. The study protocol was approved by the local institutional review board (*CPP Nord Ouest II*, Amiens, France; reference: January 22, 2015). ECs were derived from EPCs isolated from umbilical cord blood diluted 1/1 (v/v) in phosphate-buffered saline (PBS; Sigma–Aldrich). The cell suspensions were added to Ficoll solution (Eurobio) and centrifuged at 400 *g* for 30 min. Peripheral blood mononuclear cells were washed twice with PBS and then suspended in EC growth medium (EGM-2; supplemented with the Epithelial Growth Medium BulletKit; Lonza, Switzerland). Cell suspensions were seeded into 24-well plates coated with 50 μg/mL collagen I (Sigma–Aldrich). Colonies of EPCs appeared after 7 to 21 days, and ECs were expanded for 1 month before implantation. To avoid interindividual variability, ECs from the same donor were used for this study.

### Analysis of cell viability in the alginate-based hydrogel

Scaffolds (0.25 cm^2^) were prepared for cell culture experiments by washing twice with EGM-2 at 37°C (once for 1.5 h and then overnight) to neutralize the pH. BM-MSCs were labeled with a PKH67 Fluorescent Cell Linker Kit (Sigma–Aldrich) according to the manufacturer's instruction. The following numbers of cells were seeded into the scaffold, as appropriate: 5 × 10^5^ BM-MSCs only, 5 × 10^5^ ECs only, or 5 × 10^5^ BM-MSCs +5 × 10^5^ ECs together. The cells were allowed to adhere for 2 h. Fresh medium was then added to wells, and the cells were cultured for 24 or 96 h at 37°C in a 5% CO_2_ atmosphere. To evaluate cell viability and infiltration into the scaffold, medium was replaced with EGM-2 containing Hoechst 33342 (4 μg/mL) and ethidium homodimer-1 (2 μM) probes (all from Life Technologies, France) 1 h before the assay. Fluorescent microscopy was performed on an Axio Imager M2 ApoTome microscope running ZEN software (Zeiss, France).

### Animal experiments

All procedures for animal experiments were approved by the local animal care and use committee and by the French Ministry of Research (registration number: APAFIS 2625-2015110614576987 v3). All surgeries were performed under sterile conditions in the surgical suite of an animal laboratory.

Eight 8-week-old female NMRI *nude* mice (Janvier Labs, France) were housed in ventilated cabinets under controlled conditions, with *ad libitum* access to sterile chow and water. Twenty-four hours after the scaffolds had been seeded with cells (2.5 × 10^5^ MSCs only, 2.5 × 10^5^ ECs only, or 2.5 × 10^5^ MSCs +2.5 × 10^5^ ECs together), matrix constructs were engrafted subcutaneously. Mice were anesthetized by inhalation of isoflurane (induction at 4% under airflow of 1 L/min; 2% under 0.5 L/min thereafter, Isovet; Piramal HealthCare, France). Six skin incisions (5 mm) were made on the animal's back (three on the right side and three on the left), and subcutaneous pockets were created. The scaffolds were implanted (one per pocket), and four conditions were tested: (1) nonseeded (empty) scaffolds (“Alg.,” *n* = 3), (2) scaffolds seeded with BM-MSCs only (“Alg. + MSCs,” *n* = 21, three for each donor), (3) scaffolds seeded with ECs only (“Alg. + ECs,” *n* = 3), and (4) scaffolds seeded with BM-MSCs + ECs (“Alg. MSCs + ECs,” *n* = 21, three for each BM-MSC donor). The incision was then closed with surgical suture. Following surgery, animals were monitored daily for potential complications or abnormal behavior. Two months after the surgery, the animals were killed by cervical dislocation. The implants were immediately recovered and assessed for key histological and immunological parameters.

### Histological analysis

Harvested samples were embedded in Tissue-Tek O.C.T.™ (VWR) compound. Sections were prepared using a microtome-cryostat (HM 500; Microm, Germany), fixed onto glass slides, and stained with hematoxylin–eosin or Masson's trichrome. Sections were imaged under a phase-contrast microscope (Eclipse TS100; Nikon).

### Immunofluorescence analysis

Immunohistochemical staining was performed on sections fixed in acetone. Following blocking with 20% FBS, sections were incubated overnight at 4°C with the primary antibodies. On the following day, the sections were washed three times with PBS and then incubated with the conjugated secondary antibodies for 1 h at room temperature.

All antibodies were purchased from Abcam (U.K.) and BioLegend and are listed in Table 1.

**Table 1.** Antibodies Used for Immunofluorescence Analyses

Specificity	Reference
Primary antibody	
Anti-human CD90	AB 133350
Anti-mouse CD90	AB 3105
Anti-human vWF (FITC)	AB 8822
Anti-mouse CD31	AB 7388
Anti-human osteocalcin	AB 13420
Secondary antibody	
Donkey anti-rabbit (Alexa Fluor 647)	AB 150075
Chicken anti-rat (FITC)	AB 112447

FITC, fluorescein-5-isothiocyanate; vWF, von Willebrand factor.

After three washes with PBS, sections were embedded in ProLong Antifade Reagent with 4′,6-diamidino-2-phenylindole (Invitrogen). Images were acquired using an Axio Imager M2 microscope with ApoTome driven by ZEN software (Zeiss), using a 20 × /1.0 numerical aperture objective lens.

To quantify the vascular density (number of vessels per mm^2^), four sections were analyzed for each condition. Twelve images of representative fields of view were captured per section. The number of structures with a lumen surrounded by CD31- or von Willebrand factor (vWF)-positive cells in each image (0.15 mm^2^) was counted manually.

To detect MSCs and osteoblasts in the implant, three or four sections from each implant were analyzed for each condition.

### Statistical analyses

Vascular densities in three or more conditions were compared in a one-way analysis of variance with Bonferroni's test for multiple comparisons. The data are presented as median [interquartile range] (range). All statistical analyses were performed using GraphPad Prism software (version 6; GraphPad Software, Inc., San Diego, CA). The threshold for statistical significance was set to *p* < 0.05 for all tests.

## Results

### *In vitro* cell compatibility

**FIG. 1.**
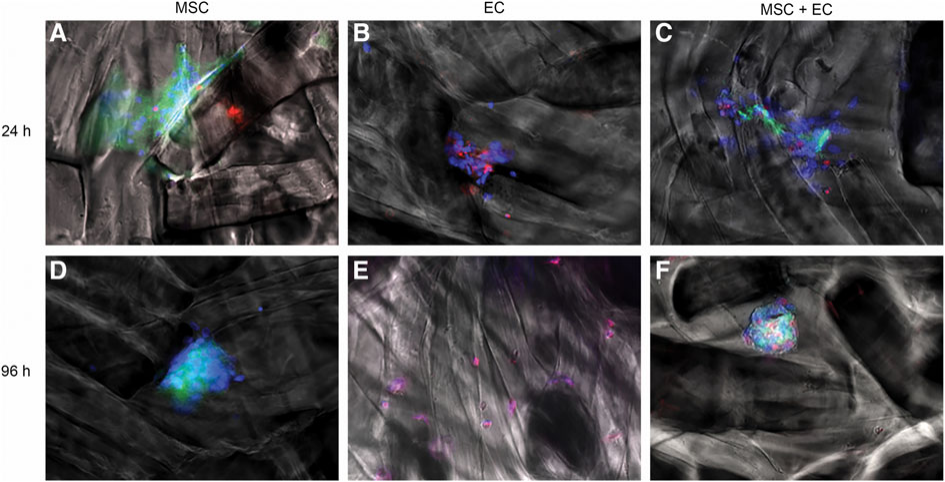
Viability of cells cultured on the alginate-based hydrogel for 24 or 96 h. The alginate-based hydrogel was seeded with MSCs alone **(A, D)**, ECs alone **(B, E),** or co-cultured MSCs and ECs **(C, F)**. The hydrogel was then cultured for 24 or 96 h. Representative fluorescence images of MSCs previously labeled with cell tracker green **(A, D)**, ECs **(B, E)**, or a co-culture of MSCs + ECs **(C F)** seeded on alginate-based hydrogel after 24 h **(A–C)** or 96 h **(D–F)**. The cell nuclei were stained with Hoechst 33342 (blue). Red fluorescence (after staining with ethidium homodimer-1) indicated the loss of plasma membrane integrity (i.e., dead cells). ECs, endothelial cells; MSCs, mesenchymal stem cells.

We seeded BM-MSCs alone, ECs alone, or BM-MSCs + ECs on the hydrogel for 24 or 96 h. After labeling with specific fluorescent lipophilic dyes, live BM-MSCs (represented in green in the figures) were detected on the surface of the hydrogel when cultured alone (Fig. 1A, D) or when cultured with ECs (Fig. 1C, F) for 24 h (Fig. 1A, C) or 96 h (Fig. 1D, F). When BM-MSCs or ECs were seeded alone on the hydrogel, they aggregated to form clusters (Fig. 1A, B, respectively). When the two cell types were seeded together, they formed mixed aggregates (Fig. 1C, F). After 96 h, live cells formed clusters (Fig. 1D, F). After 24 h of culture, the majority of BM-MSCs were viable under all conditions (Fig. 1A, C), although the number of dead cells (represented in red in the figures) increased slightly when the incubation period was longer than 4 days (Fig. 1D, F). The ECs seemed to be more sensitive to the scaffold. Some dead cells were observed after as few as 24 h in culture (Fig. 1B), and all the ECs had dead after 96 h (Fig. 1E).

### Demonstration of the scaffold's functional properties

**FIG. 2.**
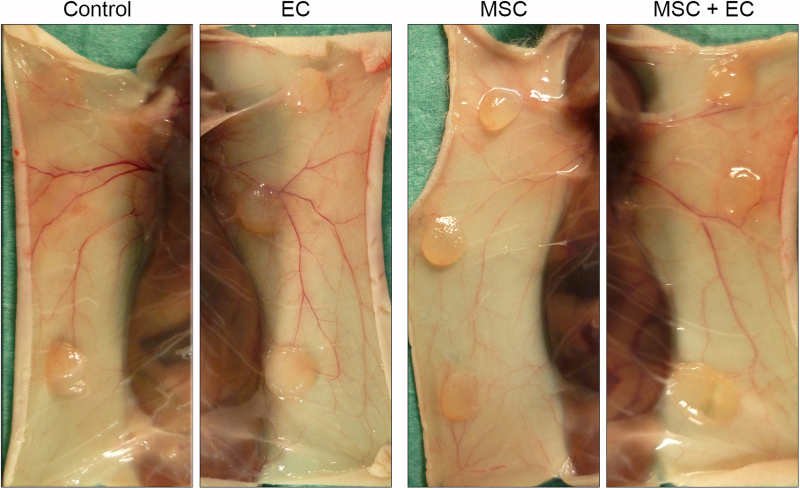
Macroscopic analysis of implanted scaffolds. Scaffolds seeded (or not) with cells and subcutaneously implanted in nude mice. The figure shows representative images of subcutaneously implanted scaffolds on the animal's back at the time of sacrifice.

We then subcutaneously implanted cell-seeded and nonseeded (control) scaffolds into *nude* mice. None of the mice died during the experiment, and we did not notice any host reactions against the implants or behavioral problems. The scaffolds were always detectable and seemed to be well integrated, as shown in Figure 2.

**FIG. 3.**
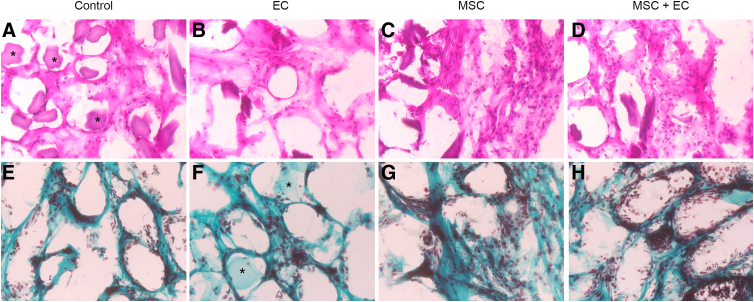
Evaluation of newly formed tissue. Representative histological images of transversal sections of ectopic implants after 2 months, after staining with H&E **(A–D)** or MT **(E–H)** (black stars indicate the scaffold). **(A, E)** Non-cell-seeded (empty) scaffolds, **(B, F)** ECs-seeded scaffolds, **(C, G)** BM-MSC-seeded scaffolds, and **(D, H)** BM-MSC- and EC-seeded scaffolds. A cellular architecture and extracellular matrix synthesis (MT staining of collagen fibrils [green] around the alginate fibers [black stars] were observed in each condition). Original magnification: × 40. BM-MSC, bone marrow-derived mesenchymal stem cell; H&E, hematoxylin and eosin; MT, Masson's trichrome.

The histological analysis revealed a cellular architecture in all control scaffolds (i.e., those not seeded with human cells), which demonstrated the host cells' ability to colonize the hydrogel (Fig. 3A). Regardless of type of cells seeded, the neotissue's morphology and structure were always the same (Fig. 3B–D). The presence of collagen synthesis in the extracellular matrix (stained in blue) was confirmed with Masson's trichrome staining. Collagen fibrils were observed around the alginate fibers (black stars) in all the groups (Fig. 3E–H).

### Demonstration of the cellularized scaffold's angiogenic properties

**FIG. 4.**
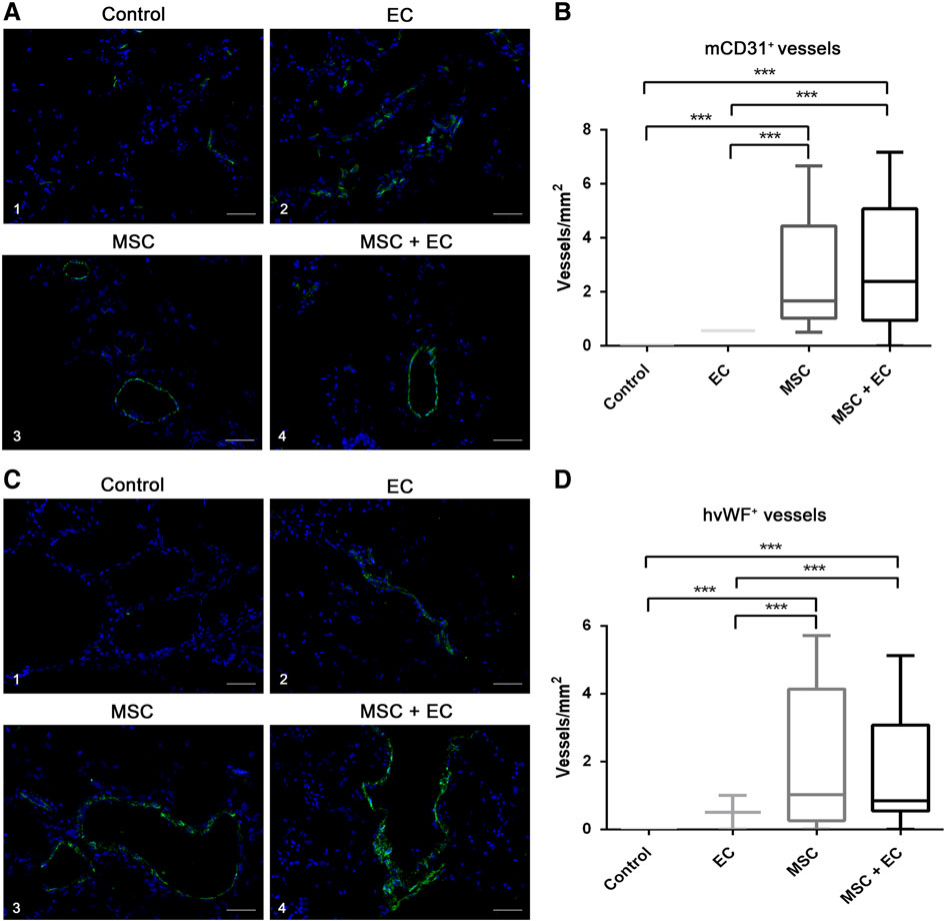
Angiogenic potential of the alginate-based hydrogel **(A)**. (**C)** The formation of vascular networks was examined in each condition: **(A.1, C.1)** non-cell-seeded (empty) scaffolds, **(A.2, C.2)** EC-seeded scaffolds, **(A.3, C.3)** BM-MSC-seeded scaffolds, and **(A.4, C.4)** BM-MSC- and EC-seeded scaffolds. Host vessels were immunostained with an AF488-conjugated anti-mouse CD31 antibody (in green) **(A.1–A.4)**. Vessels of human origin were detected by immunostaining with an FITC-conjugated anti-human vWF antibody (in green) **(C.1–C.4)**. The cell nuclei were stained with DAPI (in blue). Original magnification: × 200. (Scale bar = 50 μm). **(B, D)** Box plots of the vessel density in each condition (4 sections per group, and 12 fields of view per section). CD31-positive host vessels: **(B)** human vWF-positive vessels. **(D)** Data are presented as median [interquartile range]. The bars (whiskers) below and above the box indicate the minimum and the maximum, respectively. ***p < 0.001. DAPI, 4′,6-diamidino-2-phenylindole; FITC, fluorescein-5-isothiocyanate; vWF, von Willebrand factor.

We next examined the formation of vascular networks within the various groups of implants. To determine the origin of the cells constituting the vessels, we performed immunofluorescence analyses with a specific anti-mouse CD31 antibody (Fig. 4A.1–4) and a specific anti-human vWF antibody (Fig. 4C.1–4). Mouse CD31^+^ ECs were evidenced in all the four groups (Fig. 4A.1–4). Only a few positive cells were detected in the control group (Fig. 4A.1). There were more positive cells in the “Alg. + ECs” group, but they were not always organized into vessel-like structures (Fig. 4A.2). Conversely, implants containing BM-MSCs (“Alg. + BM-MSCs” and “Alg. + BM-MSCs + ECs” groups) contained capillary-like structures with large lumens (Fig. 4A.3 and A.4). Furthermore, the vascular density in implants initially containing BM-MSCs (whether seeded alone or together with ECs) was significantly higher than that in nonseeded implants or in implants seeded with ECs only (Fig. 4B).

The implanted human ECs' contribution to vessel formation was confirmed by the specific presence of human endothelial markers in the regenerating tissues. As expected, human vWF was not expressed in nonseeded implants (Fig. 4C.1). Human-specific vascular structures could be detected in all other groups (Fig. 4C.2, 4C.3, and 4C.4) but only in few cases in the “Alg. + EC” group. Again, we observed much larger vascular lumens when BM-MSCs were seeded in the hydrogel (compare Fig. 4C.3 and C.4 with Fig. 4C.1 and C.2). The vessel count was significantly greater in “Alg. + BM-MSCs” and “Alg. + BM-MSCs + ECs” groups (Fig. 4D). We also detected vWF^+^ vessels in the “Alg. + BM-MSCs” group. Hence, when delivered alone, BM-MSCs were able to form vessel-like tubular structures. Taken as a whole, these results suggest that BM-MSCs not only created a permissive proangiogenic environment that resulted in increased neovascularization by host cells or seeded ECs but also were able to differentiate into ECs *in vivo* and to contribute to the formation of new blood vessels.

### Osteogenic properties

**FIG. 5.**
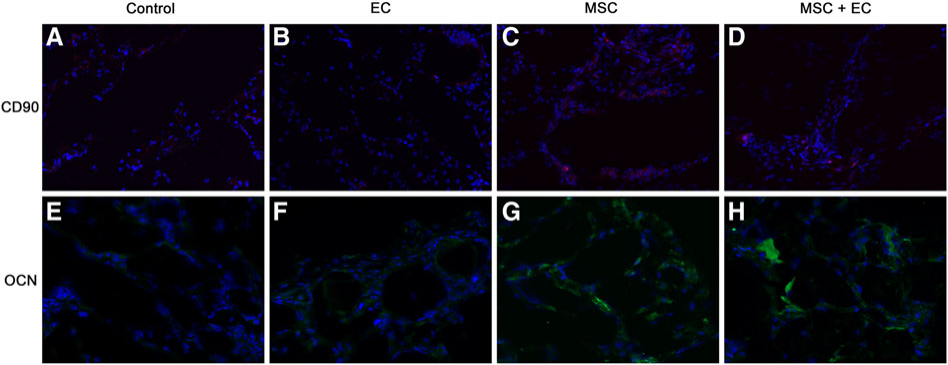
Osteogenic potential of the alginate-based hydrogel. The distribution of human CD90^+^ MSCs **(A–D)** and OCN^+^ cells **(E–H)** was evaluated within the explanted scaffolds. MSCs were immunostained with an anti-human CD90 antibody and then with an Alexa Fluor-conjugated secondary antibody (in red). OCN was immunostained with an anti-human OCN antibody and then with an FITC-conjugated secondary antibody (in green). Original magnification: × 200. OCN, osteocalcin.

We failed to observe bone formation in the implants. First, we investigated the distribution of the human CD90^+^ MSCs within the explanted scaffolds by using a specific anti-human CD90 antibody to stain cross sections. In “Alg. + MSCs” and “Alg. + MSCs + ECs” groups, human CD90^+^ cells (stained in red) were still present in the implants 8 weeks after transplantation (representative images are shown in Fig. 5C, D, respectively). We then checked whether human cells expressing osteocalcin (a late osteogenic differentiation marker) were present in the explanted scaffolds. Using a specific antibody against human osteocalcin, we observed a few positive cells (in green) in “Alg. + MSCs” and “Alg. + MSCs + ECs” groups (representative images are shown in Fig. 5G, H, respectively). Hence, it appeared that the transplanted human MSCs were present in the implants and were also able to differentiate into osteoblasts.

## Discussion

The generation of vascularized tissue constructs that mimic the structure and function of native tissues is still in early development. The slow growth of host vessels often fails to maintain the viability of the implanted constructs and prevents the latter's integration into the host tissues. Here, we demonstrated that a new alginate biomaterial has angiogenic properties *in vivo*. Host-generated vascular structures were found throughout the scaffold in all the groups of implants, although to varying extents as a function of the conditions. Only a few murine CD31^+^ ECs were observed in the control (nonseeded) group. Although more positive cells were present in the group with ECs, they did not always form vessel-like tubular structures. In contrast to the report by Bartaula-Brevik et al.,^14^ seeding human ECs seemed to have only a weak effect on the formation of a host-generated vascular network. Our observation of human vWF^+^ vessels in the “Alg. + ECs” group indicated that some transplanted ECs had been incorporated into neovessels, as already observed by Cooper and Sefton.^15^ Taken as a whole, our results suggest that (1) the addition of human ECs stimulates the migration of host ECs to the implant and (2) implanted ECs were capable to contribute to the formation of new blood vessels.

As previously described,^16,17^ we observed that EC-derived vascularization could be enhanced by the co-implantation of MSCs. This phenomenon has been well explained by the secretion of proangiogenic factors by MSCs, which enhance the migration of ECs and stem cells and thus enables blood vessel formation.^18^ The higher levels of cell retention and engraftment (human CD90-positive cells were observed inside the scaffolds seeded with human BM-MSCs explanted after 2 months) probably also justify in part the greater observed number of blood vessels, as already described by Shafiee et al.^19^ Under our experimental conditions, we observed the differentiation of implanted BM-MSCs into ECs (vWF^+^ cells were detected in scaffolds seeded with BM-MSCs alone), as reported for some *in vivo* studies.^20,21^ Our results confirm that transplanted BM-MSCs survived after transplantation and subsequently induced the formation of a vascular network via a paracrine effect and via direct integration into neovessels after differentiation into ECs. Since the transplantation of BM-MSCs alone promotes neovascularization, it might not be worth adding the ECs; this would simplify the treatment process.

We did not observe ectopic bone formation under any condition. In *ex vivo* bone tissue engineering approaches, bone formation may depend on the microenvironment.^22^ The present study was based on ectopic models of bone reconstruction, which lacked some of the bone lesion factors required for reconstruction. However, some human cells expressing osteocalcin were detected in implants seeded with BM-MSCs, demonstrating that transplanted BM-MSCs could indeed differentiate into osteoblasts within this new biomaterial *in vivo*. Even in this nonoptimal environment, the new alginate biomaterial was able to (1) maintain high levels of viability and metabolic activity in BM-MSCs and (2) create a three-dimensional environment that allowed osteoblast differentiation.

Some studies have already highlighted the value of using alginate-based hydrogels to enhance vascular regeneration. However, these strategies are often much more complex (i.e., modification of the alginate with the matrix metalloproteinase sensitive peptide Pro-Val-Gly-Leu-Iso-Gly,^3^ seeing of alginate onto a poly-caprolactone scaffold,^6^ or the addition of a proangiogenic factor^23^) or not easily adaptable to bone defect filling (i.e., an injectable formulation^4^).

## Conclusion

Our present results demonstrated the biocompatibility of a new alginate-based hydrogel, as well as the latter's potential to promote long-term cell survival and promote vascular network formation in subcutaneous implantation sites. The alginate also provides an environment that allows the osteogenic differentiation of transplanted BM-MSCs. Given the biomaterial's safety, high angiogenic capacity, high osteogenic potential, and easy-to-handle gel formulation, we hypothesize that the future clinical application of this hydrogel might accelerate wound healing and stimulate neoangiogenesis and bone regeneration.

## Abbreviations Used

Alg: alginate
BM-MSC: bone marrow-derived mesenchymal stem cell
DAPI: 4′,6-diamidino-2-phenylindole
EC: endothelial cell
EGM-2: EC growth medium
EPC: endothelial progenitor cell
FBS: fetal bovine serum
H&E: hematoxylin and eosin
MSCs: mesenchymal stem cells
OCN: osteocalcin
PBS: phosphate-buffered saline
